# Polymer Based Bioadhesive Biomaterials for Medical Application—A Perspective of Redefining Healthcare System Management

**DOI:** 10.3390/polym12123015

**Published:** 2020-12-16

**Authors:** Nibedita Saha, Nabanita Saha, Tomas Sáha, Ebru Toksoy Öner, Urška Vrabič Brodnjak, Heinz Redl, Janek von Byern, Petr Sáha

**Affiliations:** 1 Footwear Research Centre, University Institute, Tomas Bata University in Zlin, University Institute & Tomas Bata University in Zlin, Nad Ovčírnou 3685, 76001 Zlín, Czech Republic; tsaha@utb.cz (T.S.); saha@utb.cz (P.S.); 2 Faculty of Technology Polymer, Centre, Tomas Bata University in Zlin, University Institute, Centre of Polymer Systems & Tomas Bata University in Zlin, 76001 Zlín, Czech Republic; 3 Department of Bioengineering, IBSB. Marmara University, 34722 Istanbul, Turkey; ebru.toksoy@marmara.edu.tr; 4 Graphic Arts and Design, Department of Textiles, Faculty of Natural Sciences and Engineering, University of Ljubljana, 1000 Ljubljana, Slovenia; urska.vrabic@ntf.uni-lj.si; 5 Austrian Cluster for Tissue Regeneration, Ludwig Boltzmann Institute for Experimental and Clinical Traumatology, 1200 Vienna, Austria; office@trauma.lbg.ac.at (H.R.); janek.von.byern@univie.ac.at (J.v.B.)

**Keywords:** bioadhesion, biomaterials, biomedical application, healthcare system management, innovation, polymer based bioadhesive, I1, I10, I11, I18, I21, I28, H51

## Abstract

This article deliberates about the importance of polymer-based bioadhesive biomaterials’ medical application in healthcare and in redefining healthcare management. Nowadays, the application of bioadhesion in the health sector is one of the great interests for various researchers, due to recent advances in their formulation development. Actually, this area of study is considered as an active multidisciplinary research approach, where engineers, scientists (including chemists, physicists, biologists, and medical experts), material producers and manufacturers combine their knowledge in order to provide better healthcare. Moreover, while discussing the implications of value-based healthcare, it is necessary to mention that health comprises three main domains, namely, physical, mental, and social health, which not only prioritize the quality healthcare, but also enable us to measure the outcomes of medical interventions. In addition, this conceptual article provides an understanding of the consequences of the natural or synthetic polymer-based bioadhesion of biomaterials, and its significance for redefining healthcare management as a novel approach. Furthermore, the research assumptions highlight that the quality healthcare concept has recently become a burning topic, wherein healthcare service providers, private research institutes, government authorities, public service boards, associations and academics have taken the initiative to restructure the healthcare system to create value for patients and increase their satisfaction, and lead ultimately to a healthier society.

## 1. Introduction

Currently, in the 21st century, healthcare management plays an important role in focusing and aligning the myriad continuous improvements that optimize the application of bioadhesion as related to innovative biomaterials’ medical use. This article intends to reveal the importance of bioadhesive biomaterials’ application in the healthcare system. Nowadays, the application of bioadhesion is one of greatest interests for various researchers who intend to develop new biomaterials, therapies and technological possibilities, such as biomedical application. Accordingly, progressive innovation in the bioadhesion of biomaterials has trended sharply upward, and is expected to double by 2020, especially with a focus on delivering quality healthcare. Although redefining health, the World Health Organization (WHO) defined ‘health’ as a state of complete physical, mental and social wellbeing that not only considers the illness, but prioritizes the concept of value-based healthcare [1]. On the other hand, from the functional perspective, bioadhesives can be considered as an identical material, which is biological in nature and holds together for extended periods of time by interfacial forces. Essentially, it is an area of active multidisciplinary research approach, wherein engineers, scientists (including chemists, physicists, biologists, and medical experts (supportive medical), materials producers, and manufacturers combine their knowledge [2]. Finally, from the practical point of view, this article proposes some research assumptions, which state that the bioadhesion of biomaterials for redefining healthcare management is not a new concept. Its implementation has been used for several years for medical applications, such as dentistry and orthopedics, and it is now entering new fields, for example, tissue sealing and directed drug delivery systems. In addition, the said issues and solutions affect and involve healthcare delivery organizations, health plans and employers, i.e., healthcare service providers, private research institutes, government authorities and public service boards, research institutes, associations and academics. The outcome will be, in the long-term, to restructure the healthcare system, which will not only create value for patients and increase satisfaction, but it will also improve the health effects through enabling new efficiencies and lowering costs.

### 1.1. Notion of Biomaterials

Regarding the notion of “biomaterials”, it is necessary to mention that there are two significant topics that are inter-related with the concept of the word biomaterial. The first conceptual meaning of biomaterial deals with the term ‘bio’, which exemplifies, as a way of filling in the gaps where the question arises, whether we are discussing the process of taking out of life or putting into life. The second term, “material”, has a broader sense, which indicates a substance. Now the question arises of how this material can enable us to keep our life more flexible. Research shows that from the healthcare benefit point of view, several scholars have made an effort to define the term “biomaterials” and its application as well as utility in our day-to-day life. In medical science, research has shown that it has ample potential to keep our life more flexible, in that it will easily enable us to respond to altered circumstances. Although, biomaterials’ presentation in medical science did not get that recognition until the Consensus Conference on Definitions in Biomaterials Science, held in 1987. According to the European Society for Biomaterials, earlier, the term biomaterials and its medical application were not so profoundly known in the medical science, though its application was already existing [3], as the definition is a result of considered debate, which definitely has some reliability from a healthcare point of view. On the other hand, this conceptualization of biomaterials concludes that a biomaterial is “a non-viable material and its application in a medical device, is envisioned to interrelate with the biological systems” [4].

### 1.2. Overview of Bioadhesion

Bioadhesion may be defined as the binding of a natural or synthetic polymer or biological-origin adhesive to a biological substrate. When the substrate is a mucus layer, the term is known as mucoadhesion [5]. On the other hand, while referring to the application of bioadhesion in broad terms, it is necessary to mention that the terminology “bioadhesion” itself represents an extensively differentiated phenomena, as it covers the adhesive properties of both the synthetic components as well as the natural surfaces (such as cells). Furthermore, research shows that bioadhesion could also refer to the usage of bioadhesives in order to link the two surfaces together, especially in drug delivery, dental and surgical applications [6]. As such, the significance of bioadhesive biomaterial application has emerged and been recognized due to its consequences for the specific development of new biomaterials, therapies and technological products for redefining the healthcare sector.

## 2. Bioadhesion of Biomaterials

**Figure 1:**
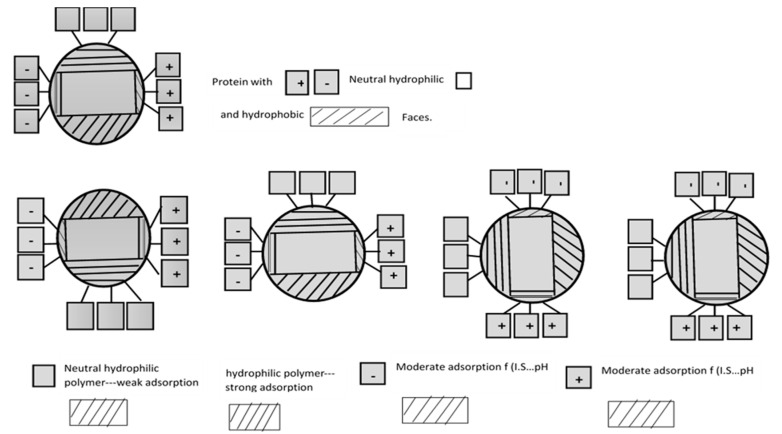
Bioadhesion of biomaterials (based on the idea from the *Bioadhesion of Biomaterials*, and Medical Devices, Springer Book [7]).

While discussing the significance of the bioadhesion of biomaterials, it is mandatory to highlight that in the contemporary world, healthcare is a fundamental issue in translational research, especially when it is innovative, as well as the fact that the bioadhesion of biomaterials application is being used in healthcare in order to fight against life-threatening diseases. In addition, over the past two decades, innovative biomaterials applications have been viewed as a significant issue in translational research in the field of regenerative medicine, where biomaterials have been extensively applied in numerous medical devices for the benefit of healthcare. In this regard, it is necessary to state that the study of biomaterials is essentially associated with the study of biocompatible materials, especially for biomedical applications, which encompasses not only the synthetic materials, such as metals, polymers, ceramics and composites, but also includes biological materials, for example proteins, cells and tissues. The below-mentioned Figure 1 shows examples of the bioadhesion of biomaterials.

On the other hand, the term bioadhesion refers to the situation wherein natural and synthetic materials stick to each other, and especially to biological surfaces. Henceforth, the application of bioadhesive polymers in healthcare emerges, specifically with the use of medical devices for the effects on the biological exterior and crossing point. In this review article, the authors attempt to prove that, from the healthcare point of view, bioadhesion’s presentation is advantageous. Considering the grafting of medical devices in the human body, it is necessary to remember that though this embedding procedure is a very useful and important aspect of healthcare, we cannot ignore the probability of high risks due to the interface for microorganisms. As implantable medical devices are the idyllic location for the growth of microbes, infections are triggered quickly by bacteria that mainly originate in the body itself. Consequently, some phases effect the bioadhesion of implantable medical devices, including surface topography, chemical interaction, mechanical interaction and physiological interactions.

Research shows that, considering these aspects, medical practitioners will likely try their best to control the medical devices through bioadhesion processes by enhancing the desirable interaction of bioadhesion and eliminating the adverse interactions. Therefore, to comprehend the debate on the bioadhesion of biomaterials in order to redefine healthcare management, it is necessary to mention some methods of the bioadhesion testing, which includes the evaluation of (a) surface roughness/surface morphology/surface topography, (b) chemical interactions, (c) physiological factors, (d) physical and mechanical effects, and (e) the contact angle and testing of biofilm formation [7,8]. In this conceptual article, focus has been placed on natural polymer-based bioadhesive biomaterials, i.e., polysaccharide/carbohydrate-based adhesives and protein-based adhesives. Carbohydrates in the form of polysaccharides are mostly available from plants (available in three different forms: cellulose, starch and natural gum), the exoskeleton of various marine animals, and/or are synthesized by some microorganisms. Cellulose is the principal structural material of the cell walls of plants. It is a homopolymer of β-d-hydroglucopyranose monomeric units that are linked via a linkage between the C-1 of the monomeric unit and the C-4 of the adjacent monomeric unit [8]. Due to the presence of the large number of hydroxyl groups, cellulose molecules readily form hydrogen bonds with other cellulose molecules so as to give highly crystalline structures, as the bonds are generally sensitive to water. These unique structural properties of cellulose are hindering its use as an adhesive itself.

As such, the future applications of these adhesives demand the modification of natural polymers so as to give components that can undergo further cross-linking to form water-insensitive bonds [8]. For example, cellulose converted to various cellulose derivatives in the form of ester and ether (e.g., cellulose acetate, carboxymethyl cellulose, hydroxyethyl cellulose, etc.) can be used in the formation of carbohydrate polymer as an adhesive. Instead, it is important to address the fact that cellulose adhesion performs at its best when connected through hydrogen bonds ranging from the macro level to the nano level. Regarding this matter, it is obligatory to mention that for knowledge about the application of these bioadhesive materials, in terms of composition, structural design and interactions with surfaces, it is crucial to expose the basic information about the biochemical and mechanical principles that are associated with the process of biological adhesion.

Similarly, protein-based bioadhesives are also recognized as one of the most significant and prolific categories of macromoecules in cells that facilitate the creation of bonding among microorganisms. In another way, it can be said that correspondingly, each protein molecule can be imagined as a polymer composed of amino acids, which are known as tiny macromolecules that contain an amine group, a carboxylic acid group and a variable side chain [9].

### 2.1. Polysaccharides-Based Adhesives

Even though cellulose, starch and gums are commercially available and used in and for adhesives, it is a challenge to establish novel adhesive polysaccharides which will be commercially available at low costs and are applicable in wet and dry states. Some interesting and praiseworthy polyssacharide-based biomaterials (bacterial cellulose, Levan, and chitosan) are stated below, which have excellent applications in the medical field.

Bacterial cellulose, as synthesized by *Acetobactor xylinum*, is a potential and promising natural polymer that has already been used quite successfully in several healthcare applications. It can be used in a wide variety of biomedical applications, from topical wound dressing to durable scaffolding that is useful in tissue engineering, and the regeneration of other tissues such as bone and cartilage. Although it was reported by Brown 1886, more attention to this biomaterial has been paid in the second half of the 20th century [10,11,12]. It is well organized in contrast to standard or plant cellulose, sometime referred as microbial cellulose. Bacterial cellulose and microbial cellulose have unique structural and mechanical properties compared to plant cellulose, but the molecular formulas (C_6_H_10_O_5_) of both bacterial and plant cellulose are the same [11,12]. Intensive study on the production of bacterial cellulose was conducted by Herstrin and Schramn (H.S.) in 1954 [13].

**Figure 2:**
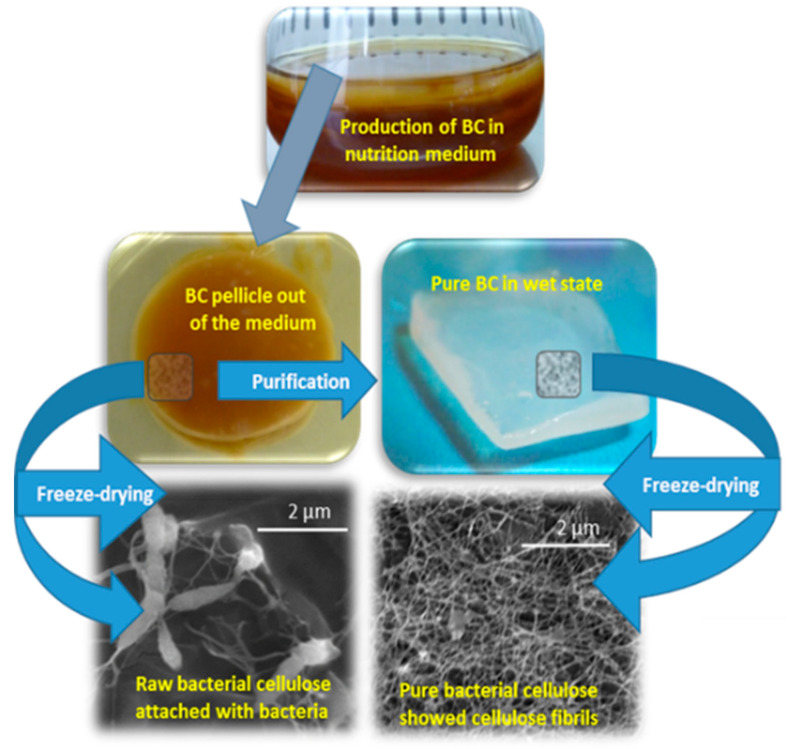
Scheme of production and purification of bacterial cellulose (BC), which exhibits the formation of cellulose fibrils by bacteria.

They established that *Acetobacter xylinum* synthesized cellulose in the presence of glucose and oxygen. Moreover, the established H.S. medium is considered as a standard nutrient medium and *A. xylinum* as a model bacterium to produce bacterial cellulose [13]. However, it is an incompetent and expensive medium for bacterial cellulose production from the present point of view. The search for cost-effective alternatives is therefore a motivation. Agro-waste-based carbon sources (coconut water, pineapple juice, etc.) are reported as an alternative nutrient medium (as fruits contain abundant sugar in the form of glucose and fructose) for the production of bacterial cellulose [14] in an economic way. At the Tomas Bata University in Zlin, the second author optimized the production conditions of bacterial cellulose using “apple juice” as a nutrient medium and “*Gluconobacter xylinus* (CCM 3611T)” as the bacterial strain [15,16,17]. The bacterial cellulose once formed is deposited on the surface of a static liquid medium (as shown in Figure 2). It is reported that the most active layer of cellulose-producing bacteria is always in contact with the air. During the process of fermentation, the older layers of cellulose are pushed down by the newly formed cellulose fibrils.

From a structural point of view, bacterial cellulose comprises a group of similar chains that are composed of d-glucopyranose units. Moreover, they are interlinked by intermolecular hydrogen bonds, which are identical in chemical composition to those of plant cellulose [17]. Such properties of BC and the lack of irregularities lead to both superior reinforcement and thermal expansion properties when used with matrix materials to form bacterial cellulose-based biocomposites [18]. From the degree of polymerization point of view, research shows that bacterial cellulose has a higher degree of purity and greater fibrousness, and the range of polymerization exists in the bacterial cellulose between 2000 and 6000. However, this relatively low stage of polymerization may limit the adhesion through interpenetrating networks or mechanical interlocking. On the other hand, in this circumstance it has been observed that in most of the cases, the adhesion in composite materials is limited to hydrogen bonding. Consequently, other applications of bioadhesion must be explored. The inter- and intra-molecular binding and/or adhesion is accomplished through hydrogen bonding and interactions with surfaces, and it is necessary to reveal the basic biochemical and mechanical principles involved in biological adhesion. According to a Vision and Technology Roadmap developed by Agenda 2020 [19], bacterial cellulose has a bright future as a renewable source of carbohydrate-based biopolymers. However, research still needs to be done for nanocellulose adhesion [19].

On the other hand, Levan is a fructan-type homopolysaccharide that is composed of fructose units joined by β-2,6 glycosidic linkages. It is widely present in nature and is produced by various microorganisms and plants from sucrose-based substrates (for a recent review, [20]), whereas microbial Levan is produced in the form of long-chained exopolysaccharides by the action of the Levan sucrase enzyme. Plant-derived Levan, instead, is shorter, and its biosynthesis takes place in the vacuoles and requires the action of several enzymes [21]. Levan stands out from other natural polysaccharides with its unusual properties such as high adhesive strength, very low intrinsic viscosity, several health benefits, and its ability to form gel alcohol and self-assembled structures. Recent efforts to associate these unique features with high-value medical applications have revived the interest in this underexplored polymer, and bring Levan into the focus of scientific and industrial interest. The applications of Levan in hair care products and whiteners, as well as its medical applications in healing wounds and burned tissue, anti-irritant, antioxidant and anti-inflammatory activities, weight loss and cholesterol control, are well documented [20].

Besides many mesophilic sources, the first extremophilic source of Levan was reported in 2009 [22]. Since then, Halomonas Levan (HL), produced by extremophilic Halomonas smyrnensis bacteria as well as its chemical derivatives, has been the subject of various high-value applications, ranging from laser-deposited bioactive surfaces to tissue engineering. These include its use in antioxidant [23] and anti-cancer [24,25] agents, as well as its suitability for the controlled delivery of peptide- and protein-based drugs [26,27] and as phosphonated HL in adhesive multilayer thin films obtained by the layer-by-layer (LbL) technique [28]. Moreover, HL was found to increase the biocompatibility and change the crystallinity in chitosan/levan/polyethyleneoxide ternary blend films [29]. It is also used as a crosslinker, and the obtained stimuli-responsive hydrogels were found to release 5-aminosalicylic acid (5-ASA) in a temperature-controlled manner [30]. Levan has also been reported as a suitable polymer for obtaining nanostructured bioactive surfaces by combinatorial matrix-assisted pulsed laser evaporation (C-MAPLE), and the obtained gradient surfaces were found to modulate the ERK signaling of osteoblasts [31,32]. Moreover, due to its high biocompatibility and heparin mimetic activity, a sulfated derivative of Halomonas Levan (SHL) has been reported to be a suitable functional biomaterial in designing engineered smart scaffolds with applications in cardiac tissue engineering [33,34]. Additionally, recently, SHL was found to not only improve the mechanical and adhesive properties of multilayered free-standing films, but also to allow myogenic differentiation, and it led to cytocompatible and myoconductive films [35]. All the above-mentioned studies make Levan polysaccharide a very promising bioadhesive for many medical applications.

Among polysaccharides, chitin and chitosan are among the most abundant natural compounds on earth, beside cellulose. Chitosan is obtained from crustaceans after the deacetylation of chitin or extraction from insects or fungi [36,37]. In view of the present scientific literature, chitosan is probably one of the most published polysaccharides. However, chitosan has not the same commercial success as cellulose. It is estimated that approximately 10 billion tons of chitin can be synthesized each year, where the main sources are crustaceans, insects, mollusks and fungi [38]. This biomaterial is still under investigation, and its adhesive properties present an industrial challenge as well as an important research area. In last decade, chitosan has gained significant attention as an adhesive biomaterial, due to its biodegradability, non-toxicity, biocompatibility and anti-microbial properties [39,40].

For the adequate adhesive properties of polymers, surface tension, ability of penetration and viscosity are the most important parameters. In a study, it was proven that the surface tension of chitosan decreases with increasing concentrations. The adhesive surface tension must be inferior at the material surface energy to obtain sufficient molecular interactions [41]. Kurtek et al. determined that 2% (*w/v*) of chitosan in a 1% (*v*/*v*) acetate solution exhibited 38.59 mN/m surface tension at the dispersive end and 1.10 mN/m in the polar part [42]. This has proven that acid-base Lewis interactions were dominating. Furthermore, chitosan with a low surface tension indicates that it is easily spread on many and different types of materials. On top of this, Bajaj et al. [43] obtained a viscosity of chitosan solution that increased with concentration, but decreased with temperature. Few researchers compared the viscosity of chitosan solutions with different molecular weights [43,44,45]. Moreover, the wide range of chitosan viscosity is an advantage in terms of its use as an adhesive. Since the adhesive viscosity depends on the application, it can be easily adapted as a chitosan solution.

Chitosan is the only cationic polysaccharide, due to the NH3⁺ group at an acidic pH [46]. The –OH, –NH_3_⁺, –NH_2_, –CH_2_OH and –NHCOCH_3_ groups of chitosan are responsible for chemical modifications intended to improve cross-linking, and consequently improve adhesiveness.

For appropriate adhesive, high tensile strength (TS) is among the important parameters. Once the material is dried, it has to achieve good mechanical properties and also good resistance to water, moisture, temperature, etc. In our study, pure chitosan films and chitosan films in blends with rice starch were prepared. The determination of the physical–mechanical properties of films has been made [40]. Films were prepared with different concentrations of chitosan, rice starch, and as plasticizers when glycerol was added [38,40,47]. The addition of glycerol led to an increase in the elasticity of chitosan films and gave high resistance to mechanical constrains. At the same time, glycerol decreased the drying time of the films, since it acted as a hygroscopic agent. The results have shown that the tensile strength of chitosan films varied from 62.3 to 64.8 MPa. These differences can be explained by the influence of ultrasound as a pretreatment and the ratio of hydrogen bonds between hydroxyl and amino groups in chitosan films [39,40,41]. The temperature of decomposition was also determined in order to characterize adhesive thermal resistance [39,47]. The thermal degradation of chitosan films was at 253 °C, and this showed that it can be used at temperatures above room temperature and even more. The analysis of chitosan paper coating films has also been made in combination with rice starch and curdlan, in different amounts of components [39,47]. Based on the results, it was determined that chitosan improved the tensile properties, decreased water vapor permeability, and improved moisture content and surface appearance, which is for the paper coating very important. Apart from this, the cross-linking of other polysaccharides, such as rice starch and curdlan, with chitosan was also evaluated for bonding applications.

The literature shows that chitosan films are very good biomaterials when used as biomedical adhesives, such as for wound healing, tissue repair, etc. [38,39,40,41,42,43]. Some commercial applications of chitosan as an adhesive are already on the market, such as Axiostat^®^ (Gujarat, India), HemConTM (Portland, OR, USA), Chitoflex^®^ PRO (Portland, OR, USA), CeloxTM (Crewe, UK) and Surgilux (Delhi, India). Chitosan has become a popular biopolymer in the medical field. Due to its unique properties among polysaccharides, it has been shown as a competitive adhesive compared to some fossil sources. Nevertheless, progress in bioadhesives should be aimed at lowering the costs and the impact on the environment. This biotechnological challenge should be focused on the environmental assessment approach, especially for developing bio-based sustainable adhesives.

### 2.2. Protein-Based Adhesives

In order to discuss the practice of biological adhesive application for medical uses, it is necessary to emphasize one of the most important aspects that needs to be taken into consideration. That is, what are the most important requirements that the organisms must fulfill? All bio-based adhesives are superbly adapted, not only in view of chemical composition, biomechanical properties and gland morphology, but also in terms of being strongly optimized for the environment and for the requirements of the organism. Aquatic adhesives, for example, perform ideally under wet conditions, but mostly show no or weak bonding ability to dry surfaces. This guarantees that prospective applications under dry conditions (i.e., as skin sealant) are less favorable for such systems.

Currently, more than 100 marine and terrestrial organisms are known to produce bioadhesives [48], some of them for 500 million years. This high variety of adhesive systems with, e.g., permanent or temporary holdfast, the ability to bond on different surfaces and with curing times from milliseconds to minutes, surely offer a broad portfolio, suitable for every desirable medical application. In the following chapter, we aim to give a short overview of existing and prospective biological adhesive systems; further details could be found elsewhere [48,49,50].

The most well-known and best-established system is certainly fibrin. Fibrin and fibrinogen are components of the blood clotting system together with thrombin, calcium ions and factor XIII. Fibrin is the most biocompatible medical sealant available today on the market [51,52]. This is in view of its biocompatibility, biodegradability, lack of heavy metals or absence of volatile organic compounds in relation to other commercial medical sealants. However, its bonding strength (approx. 0.01 MPa) is about one magnitude lower than synthetic adhesives, such as gelatin–resorcinol–formalin adhesives (approx. 0.1 MPa), which dominate today’s adhesive market [48].

One of the promising characteristics of the biological adhesives derived from marine species, such as Mytilus spec., is their curing time within seconds, strong bonding (35–75 MPa) [53] in different environments and on different surfaces (hard/soft, even Teflon^®^ [54]) and their sustainability (biocompatible, biodegradable, non-toxic, etc.). No commercial product on the market to date is able to cover such a vast application range. In Mytilus, six different L-DOPA-rich proteins (mussel adhesive protein; MAP) maintain the holdfast. The catecholic amino acid, L-DOPA (L-3,4-dihydroxyphenylalanine), is currently the best-characterized key compound in marine adhesive proteins, produced not only by Mytilus but also used by Phragmatopoma and Sabella for a permanent holdfast [55,56]. The tissue adhesive Cell-TakTM (USA) was the first example (year 1986, TM-No. 73604754) of a marine-derived sealant, based on mussel adhesive proteins only. With the technical and scientific progress within the last few years, producing L-DOPA recombinantly, technological advances in particular in the biopolymer-DOPA engineering sector have been made, shown by the increasing number of publications [57,58] and technical possibilities [59].

Snail mucus is certainly one of the most exciting and promising, but also annoying, biomaterials in the animal kingdom. Gastropods produce a temporary viscoelastic mucus (see contribution in [56,60,61]) able to bind to any sharp or smooth surfaces, even extreme anti-adhesive non-slip materials and water-coated slippery hydrogels [60]. Moreover, snail mucus is proposed to have a promoting effect on skin cell migration, proliferation, survival and antiphotoaging [62,63,64,65,66,67,68]. Consequently, snail mucus is today sold in the cosmetics sector (see Patent US 5538740 A). Moreover, its viscoelastic properties make snail mucus promising for new biomimetic medical adhesives [69]. Still little is known about the composition of this biomaterial [56] and its bonding ability on different surfaces. Additionally, different to the l-DOPA in mussels, snail mucus proteins are still not produced recombinantly; instead, the mucus is still harvested from living animals.

While most frogs and salamanders use toxic or noxious secretions as defence, some species instead use adhesives [50,70]. Upon release through epidermal glands on the body and trunk [71], the secretion of those amphibians cures immediately, enabling an irreversible and strong bonding (tensile strength >0.07 MPa, shear stress >2.8 MPa on wood) to biological (human skin) and artificial (wood, glass, metal) surfaces [55,72].

Chemical analyses show that the glue in the frog Notaden spec. and the salamander Plethodon spec. is mainly protein based (55–78% dry weight), with a high amount of water (70–90%) and a low level of sugar (0.41–0.75% dry weight) [73]. Yet there is a clear difference between the two species. In Notaden, there is a wide range (13–500 kDa) of proteins, with a few prominent protein bands (>8) and a dominant glycoprotein (Nb-1R) at 350–500 kDa [74,75]. The Plethodon glue, in contrast, contains a low range (15–120 kDa) of proteins, with a relatively high number of prominent bands (>18) and a pH from 5.0 to 8.0 [73]. Up to now, only a few biocompatibility studies for bioadhesives have been performed, probably due to their limited availability. In vitro studies on the adhesive secretions from the frog Notaden bennetti have shown that this adhesive not only shows a good cell compatibility [76], but also has a great potential for medical applications as a tissue glue [72,77,78]. Within the salamanders, some glue-producing species (i.e., *Ambystoma opacum*, *Plethodon shermani*) appear to be cell-compatible, having a probably proliferative effect on some primary cell lines [79]. The adhesive secretions of other species (*Ambystoma maculatum*, *Plethodon glutinosus*), however, have a cytotoxic effect on cell lines, making those glues less favorable candidates for potential medical applications [79].

Recently, researchers from the Ludwig Boltzmann Institute for Experimental and Clinical Traumatology have started to investigate the adhesive secretions of Chilopoda, or centipedes. The animals are known to use highly painful and lethal venoms [80], produced and secreted through glands in the forcipules (maxillipeds) to capture a wide variety of prey, including amphibians, reptiles and even mammals. As a defensive strategy, some species release on the ventral surface of each sternite [81] a fast-hardening glue droplet, which bonds strongly to glass and metal surfaces [50].

A comparison of the biochemical data of *Henia vesuviana* [82] with those of *Haplophilus subterraneus* reveals differences in the numbers and sizes of the protein bands. In the Henia glue, two major bands (12 and 130 kDa) were described [82], while in Haplophilus so far only three prominent bands, between 30 and 67 kDa, could be observed. In the adhesive defense secretion of other centipedes, cyanogenic components, such as hydrogen cyanide (HCN) and precursors (benzoyl nitrile, benzaldehyde, mandelonitrile and others) [83,84,85,86], are also present, to increase the repellent effect to predators. In the glue of Henia, such substances seem to be absent [82], and nothing is known so far of the glue of Haplophilus. A detailed and profound chemical and cytotoxic characterization of centipede glue is currently in progress, evaluating its potential as an alternative in convenient wound closure and for other tissue applications.

## 3. Bioadhesive Biomaterials’ Biomedical Applications

Bioadhesives are generally used in wound healing and hemostasis, and their use is incipient in other biomedical applications such as tissue engineering and regeneration. The incoherence between the tissue and the biomaterial is connected using the tissue adhesives in tissue regeneration [87].

Furthermore, while discussing the practical applications of bioadhesive biomaterial research in medical aspects, it is necessary to mention that over the past decade, a growing amount of attention has been paid to bone tissue engineering for research and development in bioadhesive biomaterials’ biomedical applications, and resource management, around the world to meet the societal challenges.

Accordingly, the progressive innovation in bioadhesive biomaterials has trended sharply upward, and is expected to double by 2020, especially with a focus on the application of bone tissue engineering. As such, to provide a quality healthcare service, microbially derived polysaccharides (MPs) are demanding, as they are sued for novel, multi-informant, operationally deployable, commercially exploitable and natural-origin raw materials for the production of commercially applicable products in the form of hydrogel and bio composites. These MPs are of bacterial origin (bacterial cellulose (*Acetobacter xylinum*); chitosan (*Aspergillus niger*) and Levan (*Microbacterium laevaniformans*)). Beside the applications of MP and MP-based bio-composites in the health and nano-biotechnology sectors (cell to-cell interactions, biofilm formation, and cell protection against environmental extremes), such polysaccharides are also used as thickeners, bioadhesives, stabilizers, probiotics, and gelling agents in the food and cosmetic industries, and as emulsifier, biosorbents and bioflocculants in the environmental sector.

Concerning the application of bacterial cellulose (BC), it is necessary to indicate that the application of BC has been observed in a broad spectrum, especially in different areas, such as the newspaper industry, electronics, and tissue engineering, due to its remarkable mechanical properties, conformability and porosity. This work has primarily focused on the issue of the biocompatibility of BC and BC nanocomposites and their biomedical aspects, such as surface modification for improving cell adhesion, and in vitro and in vivo studies that focus on the cellulose networks. In summation, the relevance of biocompatibility studies has also emphasized the development of BC-based biomaterials’ medical applications in bone, skin and cardiovascular tissue engineering [88].

On the other hand, as regards the biological properties’ influence on biomedical application, chitosan has many beneficial biomedical properties, such as biocompatibility, biodegradability, and no toxicity. Therefore, it has been observed that the biological activity of chitosan is closely related to its solubility. This also highlights the development and improvement of scaffolding, i.e., the support of biomaterials using a framework for regenerative medicine. Regarding biomaterials’ medical applications, it is obligatory to remark that scaffolds are one of the crucial factors for tissue engineering, such as scaffolds containing natural polymers that have recently been developed more quickly and have gained more popularity. These include chitosan, a copolymer derived from the alkaline deacetylation of chitin. In order to provide a quality healthcare nowadays, the expectations for the use of these types of scaffolds are increasing as the knowledge regarding their chemical and biological properties expands, and new biomedical applications are being investigated [89,90].

In this review article, we emphasize the intrinsic properties offered by chitosan and its medical application in tissue engineering, which proffer it as a promising substitute for regenerative medicine as a bioactive polymer. Moreover, from the application point of view, Qasim et al. [91] showed that the electrospinning of chitosan and its composite formulations for creating fibers in combination with other natural polymers is actively working in tissue engineering. It shows that the favorable properties and biocompatibility of chitosan electrospun composite biomaterials can be used for a wide range of applications [92,93].

Simultaneously, Levan is also another important and useful biomaterial, known as an Exopolysaccharide (EPS), which is mainly covered by microorganisms. These types of microorganisms are natural, nontoxic, biocompatible and biodegradable polysaccharides, which are composed of fructose units joined by β-2,6 linkages. Apart from these characteristics, Levan is also an unconventional fructose polymer produced by extremophilic microorganisms that demonstrates hydroxyl groups and that has the capability to form strong adhesive bonds with various substrates. Therefore, considering the biomedical application of Levan, research shows that it has a strong bioadhesive property. As such, bioadhesives are important devices in both biomedical and tissue engineering applications. While medical adhesives and sealants require wound healing, the robust adhesion and protection against external injure in tissue engineering is performed to ensure the improvement of biomaterial/cell interactions. From the healthcare benefit point of view, a recent study has shown that the new findings concerning Levan’s use in biomedical applications as surgical bandages and sealants and in tissue engineering mainly contribute to promoting and controlling the specific cellular responses related to their adhesion, metabolism and ideally stem cell differentiation mechanisms [94].

Apart from the above-mentioned discussion concerning some polymer-based bioadhesive biomaterials’ medical applications, it is also necessary to highlight another important Exopolysaccharide (EPS), i.e., dextran, which is excreted from the cell having bacterial origin, and is also extensively used in different kinds of biomedical applications. It is mainly useful for the following healthcare issues: magnetic separation, magnetic resonance imaging, hyperthermia, magnetically guided drug delivery, tissue repair, and molecular diagnostics [95]. Consequently, from the healthcare point of view, this research shows that currently, several technological as well as medical challenges have been determined due to the advancement of nanotechnology and to the progress of materials sciences. The usage of nanotechnology in biomedical applications has significantly shown very promising and amazing outcomes at a global scale by developing new materials with controllable and reproducible properties [96].

The protein-based adhesives materials are basically from animal sources which trigger an inflammatory response compared with human derived materials. Nowadays, various protein-based bioadhesive products are under development for clinical trials (phase III and phase IV), for example, as hemostatic sealants in cardiac surgery as vascular graft attachments, valve attachments, etc., drug delivery systems (as for example in the gastrointestinal tract, nasal delivery and ocular drug delivery), wound-healing dressings and military applications [87].

## 4. Implementation of Bioadhesive Biomaterials in Healthcare

In this contemporary age, bioadhesive biomaterials are considered as an innovative property-oriented material that is able to build an intimate relationship with the living tissue. Currently, biomaterials are revolutionizing many aspects of preventive and therapeutic healthcare that play an important role, especially during the development of new medical devices, prostheses, tissue repair and replacement technologies, drug delivery systems and diagnostic techniques. As such, due to advanced biomaterials’ promising opportunities, presently the application of biomaterials in health sectors is one of the main focuses of major research efforts around the world. Research shows that development in this field of research requires a multidisciplinary approach, whereby scientists interact with engineers, materials producers and manufacturers. On the other hand, it is necessary to mention that to face the recent challenges in healthcare management is often very demanding. Therefore, it has been observed that the required skills and resources are beyond the capabilities of a single organization, or even of a single country. Accordingly, collaborative research is thus becoming the key to achieving breakthrough results in order to bring leadership in the global marketplace [97]. “Bioadhesion of Biomaterials” covers the bioadhesion aspect of biomaterials as healthcare challenges via the research and development of effective and low-cost materials. However, their application as medical devices is limited given the degradation [7].

From the healthcare point of view, biomaterials can be demarcated as “materials that mainly clasp with some innovative properties that facilitate to emanate in immediate contact with the living tissue without eliciting any adverse immune rejection reactions.” These types of biomaterials are envisioned for usage in healthcare, especially for the purpose of the diagnosis of disease and for the treatment or for the prevention of other diseases in the human body or other animals. Additionally, it is essential to express that this condition is normally not dependent upon being metabolized for the achievement of any of its principal intended purposes or not. Equally, these devices and/or any type of biomaterials are typically used for the physical replacement of some hard or soft tissue, which has suffered any accidental damage or destruction through some pathological processes [9].

In relation to biomaterials’ applications in healthcare, it is known that biomaterials used for health purpose is not a new concept. The application of biomaterials in health issues started long ago. Although, the noticeable advancement of biomaterials application has been observed since the 1940s, but substantial development has been detected over the past 25 years, especially while applying therapeutic medical technologies and implant devices [9]. Furthermore, from the implementation of bioadhesive biomaterials’ applications in healthcare, research shows that from ancient periods, tissue adhesives’ and sealants’ applications in healthcare have renovated a lot, especially in wound management and in traumatic and surgical injuries. For example, tissue adhesives’ and sealants’ applications in healthcare are well-known for treating disorders of hemostasis (the physiological process that stops bleeding at the site of an injury while maintaining the normal blood flow circulation within the body) [98]. Instead, various biologically driven glues and synthetic adhesives are clinically utilized either for the betterment of health as an adjunct to conventional hemostats and wound closure techniques, such as suturing, or for a replacement purpose. As a result, it can be said that this kind of bioadhesive biomaterial set-up in healthcare gradually improves the ability to effectively and quickly control bleeding. Consequently, it helps in reducing the risk of complications due to severe blood loss, which is an important implementation of medical adhesives, thus making it a highly suitable tool for wound management [99]. In order to provide more vibrant information about the polymer-based bioadhesive biomaterials’ medical applications, the below-mentioned Table 1 demonstrates some examples of polymer-based bioadhesive biomaterials’ medical applications.

## 5. Redefining Healthcare Management in Relation to Bioadhesive Biomaterials’ Medical Applications

**Figure 3:**
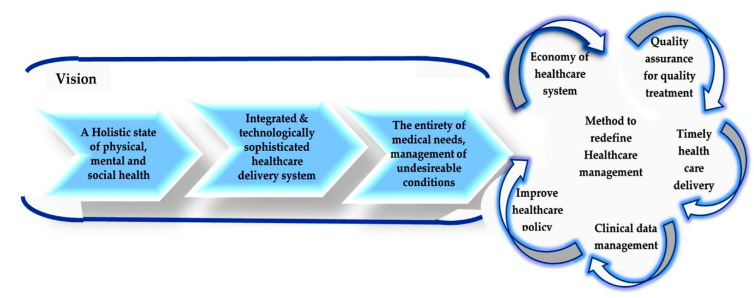
The conceptual approach of redefining healthcare management.

To address the conceptualization of “redefining healthcare management”, it is significant to discuss the idea of re-emerging “value-based healthcare” for healthy societal development. Currently, this value-based healthcare impression motivates researchers, mainly those who are interested in innovative bioadhesive biomaterial applications in healthcare due to the recent developments in their formulation. Here, engineers, scientists (i.e., chemists, physicists, biologists, and medical experts), material producers, and manufacturers combine their knowledge to reconsider all the aspects of healthcare management in order to provide and maintain the good health of a population. According to the report of the Economist Intelligence Unit [100], value-based healthcare can be considered as the formation and operation of a quality health system that explicitly prioritizes quality health products. In this regard, it is necessary to say that bioadhesive biomaterial applications in healthcare deliver quality health through integrated and technologically sophisticated heath care delivery systems. Modern healthcare also has four main principles, including the following: (i) evidence-based, patients-centered and inclusive care; (ii) community, continuous and coordinated; (iii) being ethically sound and (iv) having a regulated healthcare system [100,101,102,103]. This review article intends to describe in Figure 3 the contemporary understanding of the significance of bioadhesive biomaterials for biomedical applications in healthcare for redefining healthcare management as a novel approach.

As such, the value-based healthcare concept, i.e., to redefine the healthcare system, particularly emphasizes the proper health objective in order to increase the value. Research shows that value is generated from health consequences, which are important for the following three reasons. The presented Figure 3 illustrates that for the conceptual approach to redefining healthcare, which demonstrated the way to enhance quality healthcare as well as to maintain a programmatic approach, it is necessary to have a holistic physical, mental and social health condition or environment, a need-integrated and technologically sophisticated healthcare delivery system to provide unique patient circumstances, and care for all-inclusive patients’ medical needs, including critical and chronic disease prevention as well as the management of undesirable conditions [103].

However, to redefine healthcare, transformations must be done by both health providers and patients, as well through appropriate healthcare delivery and proper clinical data management by strengthening primary care, building integrated health systems, i.e., quality assurance for quality treatment, and implementing appropriate health payment schemes, i.e., the economy of the healthcare system that will promote the value and reduce moral hazards, enabling health information technology, and creating a policy appropriate for a healthy community [1].

**Figure 4:**
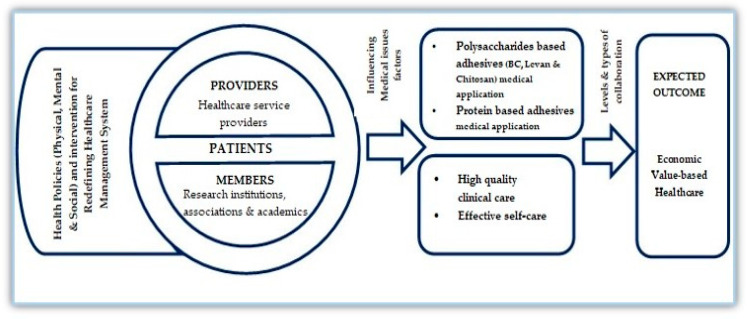
Thematic diagram of redefining healthcare management in relation to bioadhesive biomaterials (based on the idea of care management conceptual model) [101].

The conceptual framework of redefining healthcare management in relation to bioadhesive biomaterials was developed based on the idea of the care management conceptual model [101]. In this research, the main highlighted point is intended to highlight the importance of innovative bioadhesive biomaterials’ medical applications, so as to redefine all the aspects of health practice. This review article intended to raise the awareness of healthcare service providers, private research institutes, government authorities, public service boards, associations and academic initiatives to restructure the healthcare system in a way that will not only create value for patients and increase satisfaction, but it will also create a healthier society. Therefore, based on the idea of the care management conceptual model, this study develops a thematic diagram (Figure 4) to define the linkage of redefining healthcare management in relation to bioadhesives for medical applications. Figure 4 represents this connection between the healthcare service providers, patients and members, i.e., research institutions, associations and academics. This schematic diagram demonstrates the critical element of the patient in this connection, influencing medical issue factors. By including the patient element in the framework, this study considers the potential influence of patient characteristics, i.e., effective self-care and the relationships of patients with clinics/clinicians and community resources, i.e., high-quality clinical care.

To define the relation to bioadhesive biomaterials’ medical applications, it is necessary to state that biomaterials are widely used in many kinds of medical devices. The biomaterials used can be protein, metal, polymer, ceramic or composites. Similarly, bioadhesion will occur when the medical device contacts the biological surface. Figure 3 demonstrates that bacterial cellulose, Levan, and chitosan have excellent and praise-worthy applications in the medical field (already explained in an earlier part of this article).

Protein-based adhesives also play a vital role, especially when using biological adhesives for medical applications. The remarkable thing is that since primeval eras, tissue adhesives and sealant applications in healthcare have renovated a lot, particularly in wound management and in traumatic and surgical injuries. Thus, based on our previous discussion, it can be said that the processes of quality clinical care as well as patients’ effective self-care have a close connection that redefines the existing healthcare in such a way that can avoid further risks and can receive the needed preventive services. A linkage, therefore, represents the combined influence of all seven basic factors (health policies, providers, patients, members, bioadhesive biomaterials’ medical applications, quality clinical care and patients’ self-care) and their levels of collaboration that enable one to achieve the expected outcome, i.e., economic value-based healthcare for the delivery of a preventive service.

## 6. Conclusions

Finally, it can be said that this review article delivers an understanding of the consequences of the bioadhesion of biomaterials and its implications for redefining healthcare management as a novel approach, even though some research has been performed in order to describe the polysaccharides-based adhesive application at a micro level or at a nano level, which has been done for the preparation of molecularly smooth films for healthcare resolution. As such, it is necessary to continue this research in this area in order to obtain a better understanding about the adhesive interactions beyond hydrogen bonding, including mechanical interlocking, interpenetrating networks, and covalent linkages, on a fundamental level to improve the interfacial properties of thermoplastics, thermosets and biopolymers. Relating to this issue of bioadhesive biomaterials’ applications in the healthcare system, this study exposes the presentation of the progressive innovation in the bioadhesion of biomaterials. Meanwhile, today, innovative biomaterial applications tend sharply upward, and are expected to double by 2020, especially with a focus on delivering quality healthcare. While redefining health, it is necessary to mention that health consists of three main domains, namely, physical, mental, and social health, that are prioritized with a value-based healthcare concept.

The analyses revealed some important research assumptions that were predictive of both healthcare management and innovative biomaterials applications, which state that the bioadhesion of biomaterials for redefining healthcare management is not a new concept. Its implementation has been used for several years for medical applications, such as dentistry and orthopedics, and is now entering new fields, for example, tissue sealing and directed drug delivery systems. From the practical implication point of view, the results provide an important insight into the notion of involving healthcare delivery organizations, i.e., healthcare service providers, in medical science for resource management, which will help us to cope up with the socio-economic challenges of Horizon 2020. As an outcome, it is assumed that government authorities and public service boards, research institutes, associations and academics will aim to restructure healthcare systems, which will not only create value for patients and increase satisfaction, but will also improve health outcomes through enabling new efficiencies and lowering costs.

## Figures and Tables

**Table 1:** Types of polymer-based bioadhesive biomaterials’ medical applications.

Polymer-Based Bioadhesive Biomaterials	Medical Applications
Bacterial Cellulose (BC)	Drug delivery, wound dressing, implantable devices (Scaffold) and BC-based biomaterials’ medical applications in bone, skin and cardiovascular tissue engineering.
Chitosan	Tissue engineering and a promising substitute for regenerative medicine as a bioactive polymer.
Levan	Surgical bandages and sealants and in tissue engineering mainly contributing to promoting and controlling specific cellular responses related to their adhesion, and wound healing.
